# Green synthesis of zinc oxide nanoparticles utilizing extract from *Garcinia mangostana* leaves: Characterization and optimization of calcination temperature

**DOI:** 10.5455/javar.2024.k807

**Published:** 2024-09-29

**Authors:** Ridho Kurniawan Rusli, Mustofa Hilmi, Maria Endo Mahata, Ahadyah Yuniza, Zurmiati Zurmiati, Sepri Reski, Rita mutia, Cecep Hidayat

**Affiliations:** 1 Department of Nutrition and Feed Technology, Faculty of Animal Science, Universitas Andalas, Padang, Indonesia; 2 Study Program of Livestock Product Processing Tecnology, Politeknik Negeri Banyuwangi, Banyuwangi, Indonesia; 3 Department of Nutrition and Feed Technology, Faculty of Animal Science, IPB University, Bogor, Indonesia; 4 Research Center for Animal Husbandry, Research Organization for Agriculture and Food, The National Research and Innovation Agency of The Republic Indonesia, Bogor, Indonesia

**Keywords:** Calcination, *G. mangostana*, green synthesis, nanoparticle, zinc oxide

## Abstract

**Objective:**

This study aims to synthesize eco-friendly zinc oxide nanoparticles (ZnO NPs) by utilizing *Garcinia mangostana* leaf extract and assess the characteristics of ZnO NPs produced throughout different calcination temperatures (300°C, 400°C, 500°C, and 600°C).

**Materials and Methods:**

An evaluation was conducted to analyze ZnO NPs using an aqueous extract of *G. mangostana* leaf bioreductor at different calcination temperatures. The analysis involved the use of a particle size analyzer (PSA), a scanning electron microscope (SEM), energy dispersive X-ray (EDX), X-ray diffraction (XRD), and Fourier transform infrared (FTIR) spectroscopy.

**Results:**

The PSA and SEM indicated that the ZnO NPs had an average particle size ranging from 641.97 nm to 915.94 nm. Furthermore, the nanoparticles were found in both individual nanoforms and agglomerated forms. The EDX study indicated that the primary constituents of the ZnO NPs were zinc and oxygen. Additionally, the XRD examination demonstrated a distinct peak at 2θ = 36.25°, confirming the presence of a crystalline ZnO structure. The crystal size was determined to be between 40.98 nm and 46.92 nm. An FTIR spectroscopic study verified the existence of ZnO vibrations at distinct wavelengths as well as the absorption peak of the -OH functional group within the range of 3330.58 nm–3415.04 nm.

**Conclusion:**

The findings suggest that ZnO NPs produced utilizing the aqueous extract of *G. mangostana* leaves as a bioreductor can be synthesized at a temperature of 300°C, resulting in a lower particle size compared to those generated at 600°C.

## Introduction

Nanotechnology is an increasingly growing domain of scientific and technological research that has attracted considerable interest from both advanced and emerging countries. This field includes a range of applications, such as chemistry [1], pharmacy [2], food [3], biotechnology [4], animal science [5], engineering and architecture [6], and others. Utilizing nanoscale particles can augment the efficacy of active components by diminishing the necessary dosage, leading to heightened potency. There are various techniques for producing nanoparticles, such as biological, chemical, and physical processes [7]. However, the application of physical and chemical methods may require the implementation of specialized equipment and expertise and may also pose potential health hazards [8]. The green synthesis strategy, increasing popularity recently, utilizes plant extracts from several plant components, including seeds, roots, leaves, and fruits [7,9]. This strategy is considered to be more secure and is often used. The specified extracts consist of secondary metabolites or phytochemicals, including alkaloids, phenols, flavonoids, terpenoids, and so on. The compounds function as bioreductors and stabilizers during the synthesis of metal nanoparticles [10]. The green synthesis process is characterized by its environmental friendliness, simplicity, safety, cost-effectiveness, and absence of adverse effects on animals or people.

Zinc oxide (ZnO) has garnered considerable attention from researchers in the field of metal nanoparticles in recent years. ZnO nanoparticles stand out for their exceptional characteristics, including a high surface area, photocatalytic activity, and semiconductor behavior. These properties make them valuable across multiple fields, such as animal science (especially poultry nutrition), where they are utilized as feed additives. Zinc oxide nanoparticles (ZnO NPs) have been synthesized using a range of botanical extracts, including *Garcinia mangostana* [11], *Myristica fragrans* [12], *Plumbago auriculata* Lam. [13], *Origanum majorana *[2], *Cayratia pedata *[8], *Psidium guajava *[14], *green algae* [15], *Zingiber officinale* and *Allium sativum *[16], and *Viscum album* [17].

*Garcinia mangostana*, popularly known as mangosteen, is an endemic plant species found in tropical regions and belongs to the *Guttiferae* family. The aforementioned locations encompass Indonesia, Thailand, Malaysia, the Philippines, Sri Lanka, and Vietnam [11,18]. Ovalle-Magallanes et al. [19] reported that the plant’s purple-red fruit has xanthone chemicals, such as α-mangosteen. The aril, or white portion of the fruit, has a distinct flavor and scent. Moreover, chemical substances like tannins, flavonoids, and total phenols are present in plant leaves [20]. According to several studies [19,21,22], these substances possess antioxidant, antimicrobial, and antifungal qualities. Polyphenolic chemicals included in the aqueous extract of *G. mangostana* leaves are essential for the ecologically benign biosynthesis of nanoparticles. These chemicals function as capping agents, stabilizers, and reducing agents throughout the process. This study aims to synthesize eco-friendly ZnO NPs using *G. mangostana *leaf extract and assess the characteristics of ZnO NPs produced by calcination at different temperatures.

## Materials and Methods

### Materials

The* G. mangostana *(shoot) was obtained from a plantation in Koto Lua village, Padang City, in West Sumatra, Indonesia. Furthermore, the chemical compound zinc nitrate hexahydrate (Zn(NO3)26H20) was employed. In preparation for use, the glassware was thoroughly cleaned using distilled water and then dried in an oven.

### Procedure for obtaining an extract from **G. mangostana **leaves

**Figure 1.**
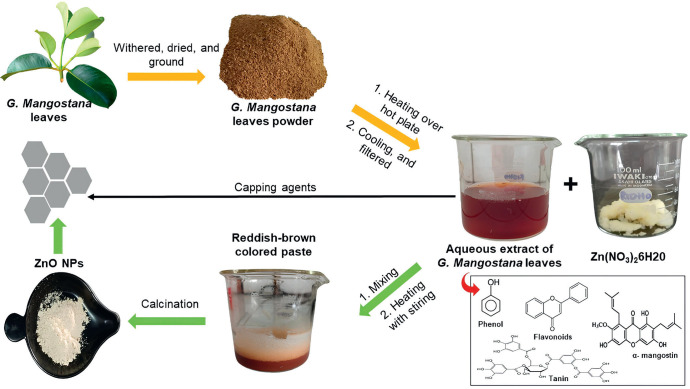
Stage of green synthesis of ZnO NPs using aqueous extract of *G. mangostana leaves.*

The acquisition of *G. mangostana leaves* has been recorded by Rusli et al. [20]. Initially (Fig. 1), the leaves underwent a thorough cleansing process using a continuous flow of water to remove any contaminants present. Afterward, they had a 24-h period of natural drying in a shaded location, followed by an additional 24-h period of drying in an oven set at a temperature of 50°C. Following the drying procedure, the leaves were crushed into a fine powder and strained through a sieve with 355 micrometer-sized holes. *Garcinia mangostana* leaf powder weighing 10 gm was combined with 100 ml of distilled water and subjected to a temperature of 50°C for 45 min. Following the cooling process, the mixture underwent two filtrations using Whatman No. 1 paper to obtain the extract. The extracted compounds were maintained at a temperature of 4°C.

### Green synthesis of nanoparticles

The process of producing the nanoparticles was extensively described by Aminuzzaman et al. [18], with adjustments implemented to the temperature at which calcination occurs. Initially, a glass beaker was filled with 50 ml of extract derived from the leaves of *G. mangostana*. The extract was after that subjected to continuous agitation while being heated to a temperature of 70°C. Furthermore, the mixture was supplemented with 4 gm of zinc nitrate hexahydrate. As the reaction progressed, the color of the combination underwent a gradual change, finally leading to the creation of a reddish-brown paste. After transferring the paste to a ceramic crucible, it was heated in a furnace at temperatures of 300°C, 400°C, 500°C, and 600°C for 2 h.

### Analysis of ZnO NPs

A particle size analyzer (PSA) called Zetasizer Nano Zs (Malvern Panalytical Ltd., The Netherlands) was used to look at the properties of ZnO NPs. The particle size distribution approach was employed for this study. To accomplish this, the liquid sample containing ZnO NPs was diluted by a factor of ten using Milli-Q water. Subsequently, the solution was subjected to centrifugation, and the resultant solution was analyzed in a cuvette. ZnO NPs were investigated for visual morphology and elemental composition using SEM analysis and energy dispersive X-ray (EDX) analysis. The inspection was carried out with a JSM-6510 device (Japan). Dispersed in methanol at a 1 mg per 20 ml concentration, the ZnO NPs displayed a size range of 0.1 nm to 10,000 nm. The surface shape, size, and structural characteristics of the ZnO NPs were investigated using X-ray diffraction (XRD). This particular work was done using the X’Pert Pro equipment (Malvern Panalytical LTD., The Netherlands). The experimental wavelength was λ = 1.5406 Å. Fourier transform infrared (FTIR) (Perkin Elmer device, USA), for investigating the surface of the ZnO NPs for functional groups. The detection occurred in the wave number range of 4,000–400 nm.

### Quantitative analysis of data using statistical methods

Characterization and optimization of the calcination temperature of ZnO NPs synthesized from environmentally friendly sources were investigated using Origin 8.5 (Origin Lab®, Northampton, USA).

## Results and Discussion

### Particle size analyzer

**Figure 2.**
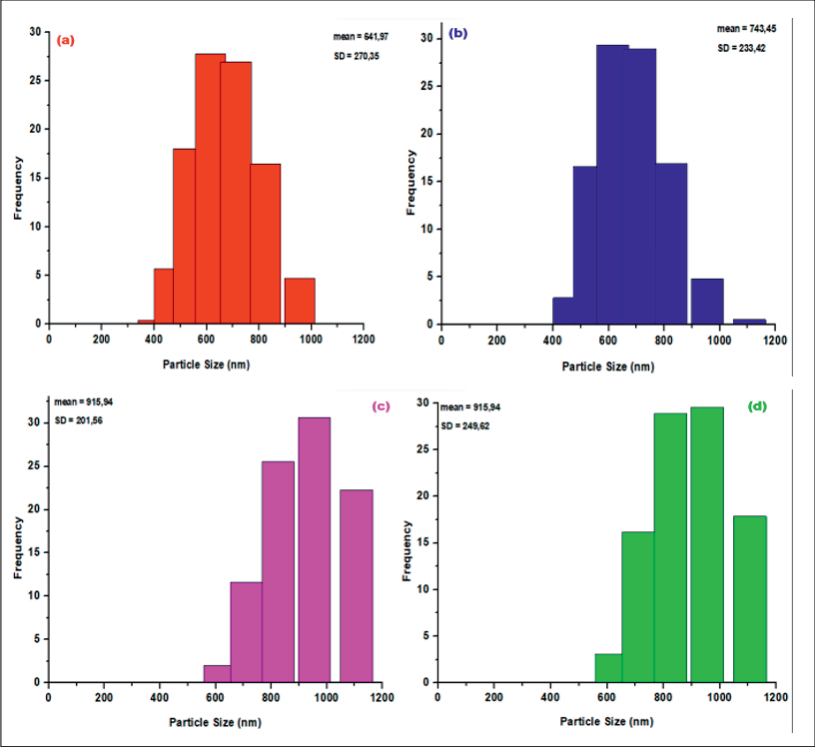
Size distribution pattern of ZnO NPs using aqueous extract of *G. mangostana leaves *at (a) 300°C, (b) 400°C, (c) 500°C, and (d) 600°C calcination temperatures.

Particle size characterization of ZnO NPs was performed using a particle analyzer, as depicted in Figure 2. The nanoparticles exhibited an average size distribution of 641.97 nm (a), 743.45 nm (b), 915.94 nm (c), and 915.94 nm (d) at five different calcination temperatures. The observed fluctuation underscores the calcination temperature’s substantial influence on both the nanoparticles’ dimensions and distribution. As illustrated in Figure 2, nanoparticles synthesized at 300°C exhibited smaller sizes and more uniform dispersion compared to those produced at higher temperatures. This observation aligns with general trends in nanoparticle synthesis, where increased calcination temperatures tend to enhance particle growth due to heightened kinetic energy and diffusion rates. Conversely, lower temperatures often favor the formation of smaller particles by restricting these processes.

Recent studies corroborate these findings. For instance, Kayani et al. [23] reported similar results, indicating that higher calcination temperatures lead to larger ZnO NPs, due to increased particle coalescence. Similarly, Sangeetha et al. [24] observed that as the temperature increased during the calcination process, the size and distribution of ZnO NPs also expanded. These findings emphasize the importance of controlling calcination temperatures to tailor nanoparticle properties for specific applications. According to the British Standards Institute’s specifications for nanoparticle dimensions as per the British Standards Institute, nanoscale materials should range from 1 to 1,000 nm [25]. The ZnO NPs produced in this study fall within this range, confirming their compliance with standard nanoparticle size requirements. Particle dimensions and dispersion are critical as they influence drug delivery efficiency, release profiles, and the overall stability of nanoparticles.

The obtained polydispersity index (PDI) values of 0.47 (a), 0.43 (b), 0.44 (c), and 0.47 (d) indicate the uniformity of the nanoparticle distribution. A PDI value close to 0 suggests a more uniform particle distribution, while values above 0.5 indicate significant heterogeneity. The consistent PDI values in this study suggest that while the nanoparticle system displays some variation, it remains relatively stable. This stability is critical for applications that require uniform particle sizes to ensure consistent performance. The present work utilized *G. mangostana* leaf extract as a reducing agent for zinc nitrate, enabling the manufacture of ZnO NPs. This natural extract not only reduces Zn²⁺ cations to Zn⁰ but also influences particle size and distribution. The presence of chemical compounds from the mangosteen extract on the nanoparticle surface affects their growth and aggregation. Previous studies have demonstrated that the properties of the reducing agent and the presence of stabilizing compounds can influence the size of nanoparticles.

As the reduction reaction progresses, the aggregation of nanoparticles is enhanced, leading to larger particle sizes. This aggregation is a result of the ongoing particle movement within the solution, which contributes to uneven particle sizes. Such behavior underscores the complexity of nanoparticle synthesis, where both chemical and physical factors must be managed to achieve the desired particle characteristics.

### Scanning electron microscope (SEM)

**Figure 3.**
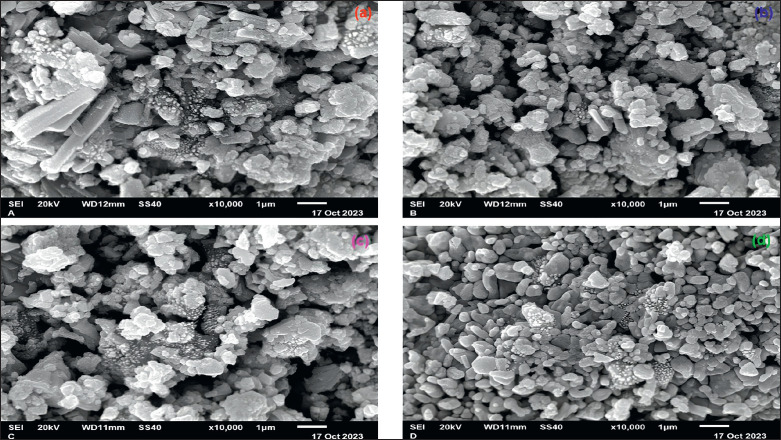
SEM of ZnO NPs using aqueous extract of *G. mangostana leaves *at (a) 300°C, (b) 400°C, (c) 500°C, and (d) 600°C calcination temperatures.

Using SEM, the appearance and dimensions of the nanoparticles produced in this study were analyzed (Fig. 3). The findings revealed that most of the ZnO NPs were at the nanoscale, though a few were still clumped together. Particles were predominantly hexagonal and spherical. The morphology of nanoparticles is a critical factor in their antimicrobial activity [15], with the hexagonal shape being especially efficient [26]. Kumar et al. [27] demonstrated that hexagonal ZnO NPs exhibit enhanced photocatalytic activity compared to spherical particles. This suggests that the hexagonal shape provides a larger surface area and more active sites for catalytic reactions. Liu et al. [28] also confirmed that the antimicrobial efficacy of ZnO NPs is significantly influenced by their shape and size, with hexagonal nanoparticles showing superior performance against a broad spectrum of bacteria [29].

The clumping observed in some of the nanoparticles synthesized in this work is a common issue that can impact their effectiveness. Recent research by Wang et al. [30] has shown that agglomeration of nanoparticles can reduce their surface area and, consequently, their reactivity and antimicrobial properties. Techniques to prevent or minimize clumping, such as surface modification and the use of dispersing agents, are actively being explored. For instance, Hameed et al. [15] demonstrated that coating ZnO NPs with biocompatible polymers can significantly reduce agglomeration and enhance their stability in various environments.

### Energy dispersive X-rays analysis

**Figure 4.**
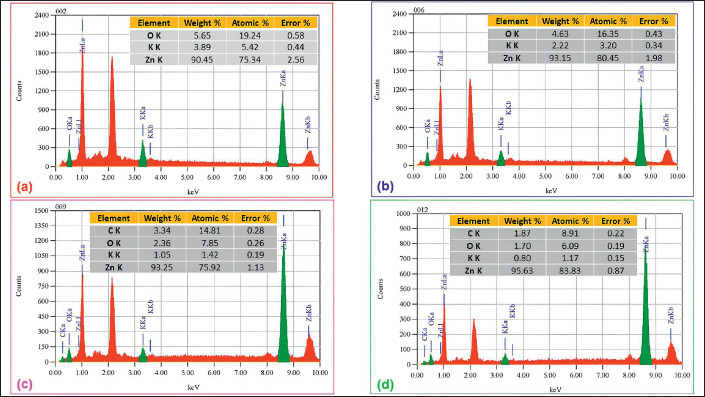
EDX analysis of ZnO NPs using aqueous extract of *G. mangostana leaves *at (a) 300°C, (b) 400°C, (c) 500°C, and (d) 600°C calcination temperatures.

An EDX analysis was used to investigate ZnO NPs isolated from an aqueous extract obtained from *G. mangostana* leaves. This procedure is depicted in Figure 4. The EDX analysis revealed substantial signals for zinc and oxygen in all samples, which showed temperature-dependent variations during calcination, indicating the presence of zinc in its oxide form. Additionally, the EDX analysis indicated that the main constituents of the ZnO NPs were zinc, with concentrations ranging from 90.45% to 95.63%, and oxygen, with concentrations ranging from 1.70% to 5.65%. In addition, the samples contained additional chemicals, including carbon and potassium.

The presence of additional elements such as carbon and potassium in the samples analyzed by EDX is consistent with findings from other studies where biological synthesis methods often incorporate trace elements from the organic materials used in the synthesis process. For instance, Wang et al. [30] explored the role of agglomeration in ZnO NPs and found that the presence of other elements can influence the dispersion and stability of the nanoparticles, which in turn affects their functional properties. These findings are important because they suggest that the synthesis method and the biological materials used can significantly impact the purity and composition of the nanoparticles.

### X-ray diffractometer (XRD)

**Figure 5.**
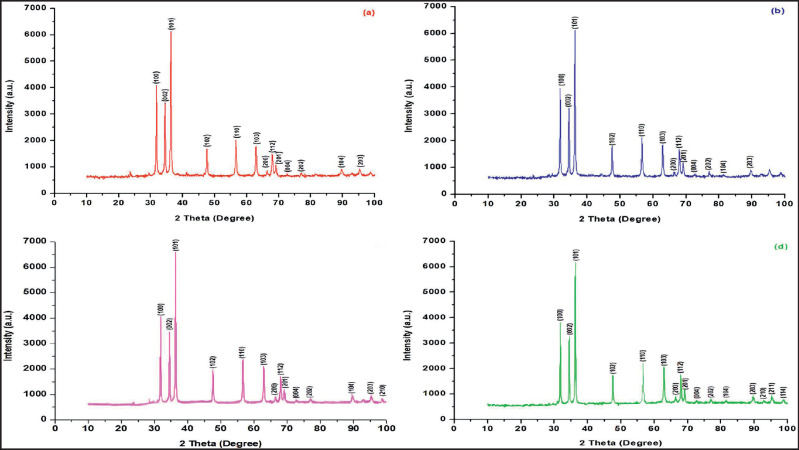
XRD Pattern of ZnO NPs using aqueous extract of *G. mangostana leaves *at (a) 300°C, (b) 400°C, (c) 500°C, and (d) 600°C calcination temperatures.

An XRD examination was performed to assess the degree of crystallinity of the ZnO samples that were subjected to various calcination temperatures (Fig. 5). The XRD patterns exhibit peak positions at 31.76, 34.42, 36.25, 47.53, 56.59, 62.85, 66.37, 67.94, 69.08, 72.56, 76.95, 81.38, and 89.60, which correspond to the (100), (002), (101), (102), (110), (103), (200), (112), (201), (004), (202), (104), and (203) crystallographic planes of ZnO. The presence of a pure and crystalline hexagonal wurtzite ZnO structure (JCPDS 36–1451) is revealed by these distinct and well-defined peaks. This discovery aligns with the outcomes documented in the existing literature on Zn nanoparticles [18]. The XRD peaks’ strength augments as the calcination temperature rises, ascribed to the combustion of the extract, resulting in the enhanced visibility of ZnO crystals [31]. Nevertheless, the strength of the distinctive ZnO peak diminished when the calcination temperature surpassed 500°C. The decrease in intensity is likely caused by the thermal breakdown of the carbon present in the ZnO-C sample, resulting in a reduction in the crystallinity of the sample. The photos demonstrate the lack of observable imperfections, suggesting the creation of ZnO nanoparticles of superior quality.

**Figure 6.**
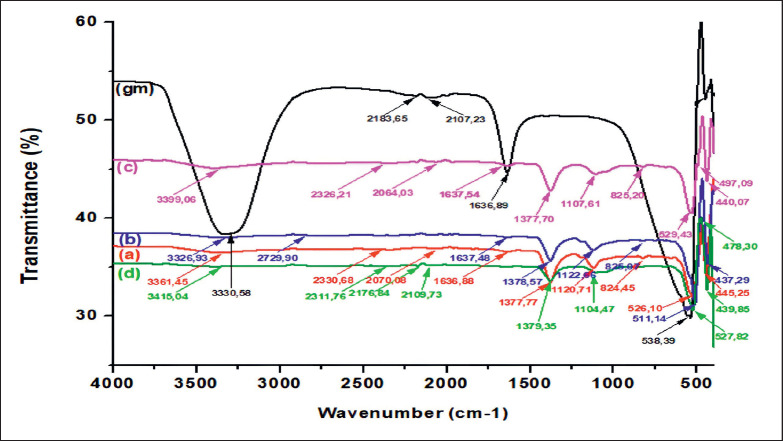
FTIR spectrum for gm = aqueous extract of *G. mangostana leaves*, a = ZnO NPs calcinated at 300°C, b = ZnO NPs calcinated at 400°C, c = ZnO NPs calcinated at 500°C, and d = = ZnO NPs calcinated at 600°C.

The equation proposed by Scherrer asserts that the numerical value of D can be expressed as the product of K and λ, divided by β and the cosine of θ. This formula includes a shape factor K of 0.9, an X-ray wavelength of 1.5406 Å denoted by λ, the total width at half maximum of the specific diffraction peak denoted by β, and Bragg’s diffraction angle denoted by θ. In this instance, the crystal size is determined by measuring the peak with the greatest intensity that corresponds to the (101) plane. The mean dimensions of each crystal are determined to be 46.92 nm for crystal a, 40.98 nm for crystal b, 46.92 nm for crystal c, and 40.99 nm for crystal d. This study emphasizes the significant discrepancy between the ZnO crystal diameters determined by XRD measurements and the particle size determined by SEM data. This is because SEM shows the general shape of the ZnO particles, while XRD gives information about the atomic crystal units inside each particle.

### Fourier transform infrared

The synthesized materials were analyzed using FTIR spectroscopy to identify the functional groups of interest. Figure 6 illustrates the FTIR spectra of the water-based extract of *G. mangostana* leaves and ZnO NPs that underwent calcination at temperatures 300°C, 400°C, 500°C, and 600°C. The similarity between the graph of ZnO nanoparticles and the aqueous extract of *G. mangostana* leaves is readily apparent. The results suggest that the phytochemical constituents present in the mangosteen leaf extract were also detected in ZnO nanoparticles. An analysis of the functional groups present in the extract of mangosteen leaves and ZnO NPs, subjected to varying temperatures of 300°C, 400°C, 500°C, and 600°C, is shown in Table 1. The chemical components in the FTIR spectra were determined using the database described in reference [32].

When synthesizing the ZnO NPs, the spectral peaks of the methanol extract of *G. mangostana* leaves showed significant variations. More specifically, the peaks observed at 2,183.65 cm^-1^ and 2,107.23 cm^-1 ^were no longer present in the synthesized ZnO NPs. The disappearances could be ascribed to the phytochemicals found in the extract, which function as reducing agents for the zinc ions. In addition, many peaks exhibited changes in position, such as the transition from 3,330.58 cm^-1^ to a range of 3,326.93–3,415.04 cm^-1^, 1,636.89 cm^-1^ to a range of 1,636.88–1,637.54 cm^-1^, and 538.39 cm^-1^ to a range of 511.14–529.43 cm^-1^. The observed shifts indicate the presence of phytochemical components in the extract, which serve as reducing agents, stabilizers, and carriers of ZnO nanoparticles [2,11,31]. The hydroxyl (-OH) group is involved in the oxidation-reduction process of metal ions [15], while the carboxylate (-COO-) group stabilizes nanoparticles [33]. However, the distinct peak at 439.85–497.09 cm^-^1 was only observed in ZnO nanoparticles that were synthesized at elevated calcination temperatures. The presence of this peak can be ascribed to the vibrations of zinc-oxygen bonds, which have been previously seen at frequencies ranging from 434 to 500 cm^-^1 in investigations conducted by Chan et al. [11], Hameed et al. [15], and Kayani et al. [23]. Due to interatomic vibrations, the absorption maxima of metal nanoparticles in oxides and hydroxides typically occur at wavelengths below 1,000 cm^-1^. The observed variations in the harmonic vibrations of the Zn-O bond at various calcination temperatures can be ascribed to the interplay between the functional groups present in the plant extracts. The identified functional groups of ZnO NPs, including phenols, alcohols, amines, alkenes, and carboxylic acids, play a crucial role in reducing, stabilizing, and capping the nanoparticles produced by the process under investigation [2].

**Table 1.** FTIR peak for assignment of aqueous extract of *G. mangostana leaves* and ZnO nanostructures calcinated at different temperature.

No	gm	a	b	c	d	Functional groups
1	3,330.58	3,361.45	3,326.93	3,399.06	3,415.04	O-H Stretching, N-H Stretching
2	−	−	2,729.90	−	−	C-H aldehyde; O-H Carboxylic Acids
3	−	2,330.68	−	2,326.21	2,311.76	P-H Phospine; Si-H Silane
4	2,183.65	−	−	−	2,176.84	Si-H Silane; N=C=O Isocyanates; N=C=S, Isothiocyanates, N=C=N Diimides, N_3_ Azides, C=C=O Ketenes
5	2,107.23	−	−	−	2,109.73	C C alkyne
6	−	2,070.08	−	2,064.03	−	−
7	1,636.89	1,636.88	1,637.48	1,637.54	−	C=C Alkene; C=O Amides; NH_2 _Amines
8	−	1,377.77	1,378.57	1,377.70	1,379.35	S=O Sulfone
9	−	1,120.71	1,122.66	1,107.61	1,104.47	C-O Alkohol, C-O Carboxylic acids; C-N Amines, P=O Phosphine oxide
10	−	824.45	825.07	825.20	−	C-C Alkane, NH_2_ & N-H Amines; S-OR Esters
11	538.39	526.10	511.14	529.43	527.82	S-S Disulfide
12	−	−	−	497.09	478.30	Zn
13	−	445.25	437.29	440.07	439.85	Zn

Note: gm = aqueous extract of *G. mangostana leaves*, a = ZnO NPs calcinated at 300°C, b = ZnO NPs calcinated at 400°C, c = ZnO NPs calcinated at 500°C, and d = ZnO NPs calcinated at 600°C.

## Conclusion

ZnO NPs are characterized using various techniques, including PSA, SEM, EDX, XRD, and FTIR spectroscopy at different calcination temperatures (300°C, 400°C, 500°C, and 600°C). As a bioreactor, an aqueous extract of *G. mangostana* leaves was used in this study to show that ZnO NPs can be made at 300°C. Furthermore, the size of the nanoparticles is less than that produced at 600°C. More research (*in vitro* study) needs to be done to find out how well ZnO NPs kill pathogenic bacteria like *Salmonella* sp., *Escherichia coli*, and *Staphylococcus aureus*.

## List of Abbreviations

EDX, energy dispersive X-rays; FTIR, Fourier transform infrared; PDI, polydispersity index; PSA, particle size analyzer; SEM, scanning electron microscopes; XRD, X-ray diffraction; ZnO NPs, Zinc oxide nanoparticles.
